# Supplementary material for: Programed Thermoresponsive Polymers with Cleavage-Induced Phase Transition
Source: Molecules. 2022 Sep 18;27(18):6082. doi: 10.3390/molecules27186082 (PMC9501266; doi:10.3390/molecules27186082)
Supplement: Supplementary file 1 [file molecules-27-06082-s001.zip › molecules-1923642-supplementary.pdf]

[Supporting Information]

Programed Thermoresponsive Polymers with Cleavage-Induced Phase Transition

*Yukiya Kitayama[1,2], Yasumichi Yazaki[1], Junya Emoto[1], Eiji Yuba[1,2], Atsushi Harada[1,2]**

1: Department of Applied Chemistry, Graduate School of Engineering, Osaka Prefecture University, 1-1, Gakuen-cho, Naka-ku, Sakai, Osaka 599-8531, Japan

2: Department of Applied Chemistry, Graduate School of Engineering, Osaka Metropolitan University, 1-1 Gakuen-cho, Naka-ku, Sakai, Osaka 599-8531, Japan

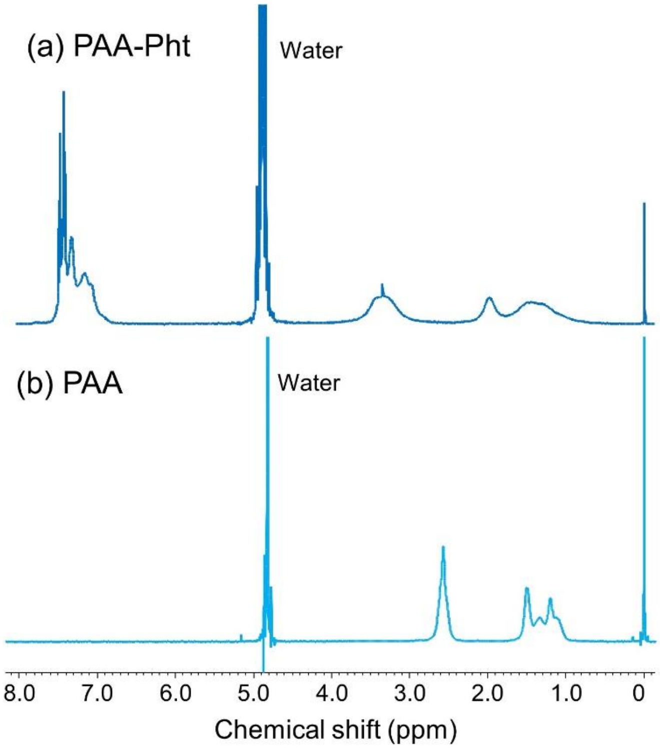

Figure S1. ^{1}H-NMR spectra of PAA-Pht (a) and PAA (b).

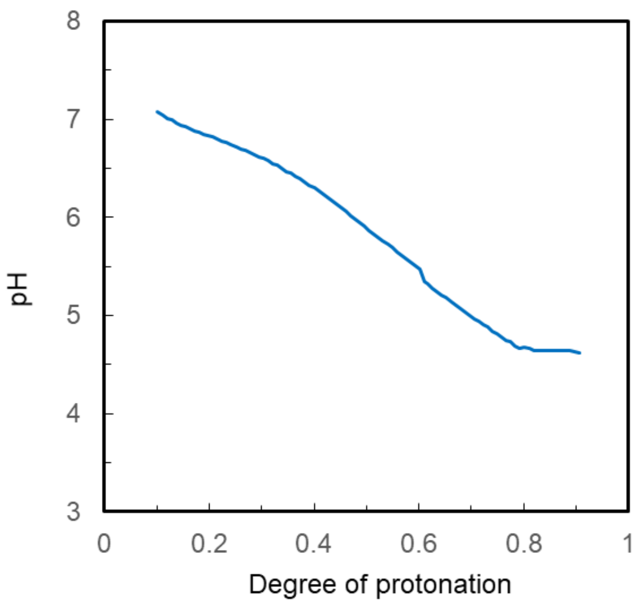

Figure S2. Acid-base titration curve of PAA-Pht.
